# Supplementary material for: Plasma circulating cell-free mitochondrial DNA in depressive disorders
Source: PLoS One. 2021 Nov 4;16(11):e0259591. doi: 10.1371/journal.pone.0259591 (PMC8568274; doi:10.1371/journal.pone.0259591)
Supplement: Attachment — Submitted filename: Response letter oct20_final.docx [file pone.0259591.s002.docx]

Comment from editor:

The essence of the other discussions made based on the comments by the referee 2 should also be incorporated into the manuscript, i.e., statistical power (point 1), handling time (point 2).

Authors response: Thanks for this comment. We have now added, to the discussion section (p17):

“The current study was originally designed to test another primary hypothesis, namely the relationship between genetic variants and suicidal behavior. Thus, we did not, *a priori*, power the study with the main intent to investigate differences in ccf-mtDNA between patients and controls. Although this might be considered a weakness of the study, our previous experiences using the same biomarker assay in other MDD/control cohorts (with substantially smaller sample size than the present study) showed effect sizes ranging between Cohen’s d of 0.9 (9) to Cohen’s d >2 (8). Therefore, we believed that the current sample size would be large enough to detect a significant group difference between patients and controls.”

In the results section (page 12) we report associations between ccf-mtDNA and handling time and we have now also added one sentence about this in the Discussion (page 16-17):

“Sample storage time in freezer differed significantly between patients and controls, but was not significantly correlated with ccf-mtDNA. Moreover, the main group effects remained significant even after taking this factor into account, making it unlikely that storage time confounded our results.”
